# Neutralizing Antibody and T-Cell Responses against SARS-CoV-2 Wild-Type and Variants of Concern in Chronic Obstructive Pulmonary Disease Subjects after ChAdOx-1/ChAdOx-1 Homologous Vaccination: A Preliminary Study

**DOI:** 10.3390/vaccines10122176

**Published:** 2022-12-18

**Authors:** Warawut Chaiwong, Nuchjira Takheaw, Witida Laopajon, Supansa Pata, Pilaiporn Duangjit, Juthamas Inchai, Chaicharn Pothirat, Chaiwat Bumroongkit, Athavudh Deesomchok, Theerakorn Theerakittikul, Atikun Limsukon, Pattraporn Tajarernmuang, Nutchanok Niyatiwatchanchai, Konlawij Trongtrakul, Kantinan Chuensirikulchai, Passaworn Cheyasawan, Chalerm Liwsrisakun, Watchara Kasinrerk

**Affiliations:** 1 Division of Pulmonary, Critical Care, and Allergy, Department of Internal Medicine, Faculty of Medicine, Chiang Mai University, Chiang Mai 50200, Thailand; 2 Division of Clinical Immunology, Department of Medical Technology, Faculty of Associated Medical Sciences, Chiang Mai University, Chiang Mai 50200, Thailand; 3 Biomedical Technology Research Center, National Center for Genetic Engineering and Biotechnology, National Science and Technology Development Agency at the Faculty of Associated Medical Sciences, Chiang Mai University, Chiang Mai 50200, Thailand

**Keywords:** SARS-CoV-2, variants of concern, COVID-19 vaccine, adenovirus-vectored vaccine, COPD, elderly

## Abstract

Data on immunogenicity of adenovirus-vectored vaccine in chronic obstructive pulmonary disease (COPD) patients is limited. Therefore, we aimed to determine the humoral and cellular immune responses after homologous ChAdOx-1 vaccination in subjects with COPD. COPD subjects and age- and sex-matched healthy elderly receiving ChAdOx-1 homologous vaccination were included. The levels of neutralizing antibodies (NAb) and specific CD4 and CD8 T-cell responses against SARS-CoV-2 wild-type (WT) and variants of concern (VOCs: Alpha, Beta, Delta, and Omicron) were measured. Eight COPD patients were matched with eight control participants. After vaccination for 4 and 12 weeks, % inhibition of NAb against Alpha, Beta, and Delta in both groups were comparable and significantly higher than baseline. The percentage inhibition of NAb at the 12th week was significantly dropped from the 4th week in each group. The NAb against the Omicron variant, however, were much lower than Alpha, Beta, Delta variants. The increasing trend in the number of CD4 T-cells producing TNF-α, IFN-γ, IL-10, and FasL upon stimulation with spike peptides of WT and VOCs was observed in COPD patients compared to the healthy group. These responses were not observed in CD8 T-cells. Homologous ChAdOx-1 vaccination could induce comparable NAb against the SARS-CoV-2 WT, Alpha, Beta, Delta, and Omicron variants between COPD and healthy elderly. The CD4 T-cell responses did not differ between COPD patients and healthy control.

## 1. Introduction

More than 600 million people worldwide have been infected with severe acute respiratory syndrome coronavirus 2 (SARS-CoV-2), which is the cause of Coronavirus disease 2019 (COVID-19), since December 2019. Chronic obstructive pulmonary disease (COPD) is one of the risk factors for more severe diseases [1]. Current evidence showed that COPD subjects have increased pulmonary expression of angiotensin-converting enzyme 2 (ACE2) and transmembrane serine protease 2 (TMPRSS2) in lung tissues, which may predispose patients to an increased risk of worse outcomes from COVID-19 [2]. The latest GOLD guidelines suggested that COPD subjects were at risk of poor COVID-19 outcomes and should be prioritized for a vaccination with COVID-19 vaccines [3]. Unfortunately, subjects with COPD might have a limited response to the vaccine. From the inactivated influenza vaccination model, subjects with COPD had a lower antibody response to the influenza vaccine in contrast with healthy non-COPD controls [4,5,6]. COPD patients also had impaired B-cell and T-cell responses to the influenza vaccine in comparison with healthy controls [6]. For COVID-19 vaccines, a previous study showed that in subjects with chronic pulmonary disease, with established humoral immune responses to two doses of BNT162b2 mRNA COVID-19 vaccination, the reactions were lower than healthy controls [7]. However, subjects in the control group of this study were significantly younger than the study group. In addition, this study included heterogeneity of respiratory diseases, not only COPD patients. Therefore, results on COPD patients were inconclusive. A study published by Pelletier et al. showed that after receiving the second dose of vaccine (mostly mRNA vaccine), subjects with COPD tended to have a lower antibody response against S protein compared to healthy controls but not significantly [8]. However, another study demonstrated that, despite higher age, COPD patients had comparable anti-S antibody responses with healthy controls [9]. Therefore, the humoral and cellular immune responses against SARS-CoV-2 wild-type (WT) and variants of concern (VOCS) after receiving COVID-19 vaccination in subjects with COPD are still controversial.

In Thailand, the adenovirus-vectored vaccine (ChAdOx1) is one of the COVID-19 vaccines authorized for emergency use, and subjects with COPD are indicated as a high-risk group requiring early vaccination. However, this vaccine had limited data on immunogenicity in patients with COPD. In this study, a project of a prospective study on immunologic responses to ChAdOx1/ChAdOx1 homologous COVID-19 vaccine in the healthy elderly with controlled underlying diseases including COPD was performed. The objective of this sub-study was to compare the levels of neutralizing antibody (NAb), and specific CD4 and CD8 T-cell responses against SARS-CoV-2 WT and VOCs between vaccinated COPD patients and vaccinated healthy elderly subjects after receiving the second dose of ChAdOx-1/ChAdOx-1 homologous vaccination.

## 2. Materials and Methods

### 2.1. Materials

For neutralizing antibody assay, the cPass SARS-CoV-2 neutralization antibody detection kit for WT and VOCs was purchased from GenScript Biotech, NJ, USA. For spike (S)-specific T-cell response assay, PepTivator^®^ SARS-CoV-2 Prot_S Complete (peptide pools 15mer sequences with 11amino acids overlap covering the full length of WT spike proteins) were purchased from Miltenyi Biotech (Bergisch Gladbach, Germany). For determining T-cell responses to S peptide of VOCs, PepTivator^®^ SARS-CoV-2 Prot_S B.1.1.7 Mutation Pool, PepTivator^®^ SARS-CoV-2 Prot_S B.1.1.7 WT Reference Pool, PepTivator^®^ SARS-CoV-2 Prot_S B.1.351 Mutation Pool, PepTivator^®^ SARS-CoV-2 Prot_S B.1.351 WT Reference Pool, PepTivator^®^ SARS-CoV-2 Prot_S B.1.617.2 Mutation Pool, and PepTivator^®^ SARS-CoV-2 Prot_S B.1.617.2 WT Reference Pool were purchased from Miltenyi Biotech. For immunofluorescence staining, FITC-labeled anti-CD3 monoclonal antibody (mAb), PerCP-labeled anti-CD4 mAb, BV785-labeled anti-CD8 mAb, PECy7-conjugated anti-human IFN-γ mAb, PECy7-conjugated anti-TNF-α mAb, BV421-conjugated anti-Fas ligand (FasL) mAb, BV421-conjugated anti-IL-17A mAb, PE-conjugated anti-human cytokines (IL-4, IL-10, IL-17A) mAbs, and fluorochrome-conjugated isotype-matched control mAbs were purchased from BioLegend (San Diego, CA, USA). Brefeldin A and monensin were purchased from Sigma-Aldrich (Saint Louis, MO, USA). Saponin and paraformaldehyde were purchased from Riedel de Haen (Seelze, Germany). 

### 2.2. Study Design and Participants

The second analysis of the prospective study in healthy elderly with controlled underlying diseases was conducted. All subjects were recruited at Chiang Mai University Hospital between June 2021 and December 2021. COPD patients, diagnosed when they had a smoking history > 10 pack-years, compatible clinical manifestations and diagnostic spirometry results with the post-bronchodilator ratio of forced expiratory volume in the first second (FEV_1_)/forced vital capacity (FVC) < 0.7 according to the Global Initiative for the Diagnosis, Management, and Prevention of Chronic Obstructive Lung Disease (GOLD) 2020 [3], were included. All spirometry results in the past year were reviewed and selected according to American Thoracic Society (ATS)/European Respiratory Society (ERS) criteria [10]. The spirometric predicted values were calculated using Knudson’s reference equations [11]. All COPD patients had to have no exacerbation within three months before receiving the vaccine. The age-, sex-, and vaccine regimen-matched healthy elderly were also included in our study. The exclusion criteria for both COPD and healthy elderly were history of infection with SARS-CoV-2, history of contact with COVID-19 patients within two weeks before enrollment, history of receiving other vaccines against SARS-CoV-2, history of receiving the attenuated live vaccine in the past 28 days, history of receiving inactivated or subunit vaccines in the past 14 days, history of allergy to any study vaccine component, uncontrolled underlying disease, e.g., diabetes, cardiovascular disease, pulmonary disease, an end-stage renal disease requiring dialysis, cirrhosis, and immunocompromised host or receiving immunosuppressive agents. All subjects received ChAdOx-1/ChAdOx-1 homologous vaccinations. This study was approved by the ethical committee of the Faculty of Medicine, Chiang Mai University (Institutional Review Board (IRB) approval number: MED-2564-08247, date of approval: 16 June 2021) and filed under the Clinical Trials Registry (Study ID: Study ID: TCTR20210822002, date of approval: 22 August 2021) in compliance with the Declaration of Helsinki. Before enrollment, written informed consent was obtained from all subjects.

### 2.3. Study Procedures

Both COPD and age-, sex-, vaccine regimen-matched healthy elderly subjects were included in the analysis. The demographic data were collected including age, sex, height, body weight, body mass index, smoking status, and underlying diseases. For COPD subjects, the inhaled medications and spirometry results were also reviewed. Blood samplings were taken before receiving the first vaccination and at 4 and 12 weeks after the second dose of vaccination. Blood samples of all participants were processed for testing the level of NAb. Blood samples four weeks after receiving the second dose of vaccination were used for the determination of CD4 and CD8 T-cell responses. 

### 2.4. Assay for Neutralizing Antibodies 

SARS-CoV-2 neutralization test for WT, Alpha, Beta, Delta, and Omicron variants was performed using the cPass SARS-CoV-2 neutralization antibody detection kit (Genscript Biotech). To determine SARS-CoV-2 NAbs, plasma, positive and negative controls were diluted with sample dilution buffer and pre-incubated with the horseradish peroxidase (HRP) labeled receptor binding domain (RBD) proteins of WT, Alpha B.1.1.7 (mutation site at N501Y), Beta B.1.351 (mutation site at E484K, K417N and N501Y), Delta B.1.617.2 (mutation site at L452R and T478K), Omicron BA.2 (mutation site at G339D, S371F, S373P, S375F, T376A, D405N, R408S, K417N, N440K, S477N, T478K, E484A, Q493R, Q498R, N501Y, Y505H), or Omicron BA.4&BA.5 (mutation site at G339D, S371F, S373P, S375F, T376A, D405N, R408S, K417N, N440K, L452R, S477N, T478K, E484A, F486V, Q498R, N501Y, Y505H) at 37 °C for 30 min. The mixture was then added to the capture plate which was pre-coated with the human angiotensin-converting enzyme 2 (ACE2) and incubated at 37 °C for 15 min. The unbound HRP labeled RBD proteins were removed by washing. TMB (3,3′,5,5′-Tetramethylbenzidine) substrate solution was added followed by the stop solution. The absorbance of the final solution was read at 450 nm with a microtiter plate reader. The % inhibition was calculated from O.D. at 450 nm as follows: [1—(O.D. value of sample/average O.D. value of negative control from the corresponding strain)] × 100. The 30% signal inhibition was used as the cutoff for SARS-CoV-2 NAb detection according to the manufacturer (GenScript Biotech).

### 2.5. Assay for CD4 and CD8 T Responses 

Peripheral blood mononuclear cells (PBMCs) were isolated from heparinized blood by Ficoll-Hypaque gradient centrifugation. The isolated PBMCs were stimulated with S peptides, consisting of SARS-CoV-2 Prot_S Complete (all functional domains of S protein of WT), Prot_S B.1.1.7 Mutation Pool, B.1.617.2 Mutation Pool, or Prot_S B.1.617.2 Mutation Pool (covering selectively the mutated regions in S protein of Alpha, Beta, and Delta variants, respectively), or Prot S B.1.1.7 WT Reference Pool, Prot_S B.1.617.2 WT Reference Pool, or Prot_S B.1.617.2 WT Reference Pool (homologous peptides of the WT sequence of Alpha, Beta, and Delta variants mutation pool, respectively), according to manufacturer protocol (Miltenyi Biotech). Briefly, PBMCs were stimulated with indicated peptide pools and incubated in a 5%CO_2_ incubator at 37 °C for two hours. The protein-releasing inhibitors, brefeldin A (1 µg/mL) and monensin (1 µM) were then added and continuously incubated for four hours. After incubation, cells were harvested and washed two times. Cells were fixed with 4% paraformaldehyde for 15 min at room temperature and were washed twice. For Fc receptor blocking and permeabilization of cells, 0.1% saponin containing 10% human blood group AB serum was added and incubated at 4 °C for 30 min. To determine spike-specific T-cell response, cells were intracellularly stained with cocktail antibodies for analysis of TNF-α, IFN-γ, IL-4, IL-10, IL-17A, and FasL using fluorochrome-conjugated specific mAbs. To identify the T-cell subset, the membrane surface markers including BV785-conjugated anti-CD8 mAb, PerCP-conjugated anti-CD4 mAb, and FITC-conjugated anti-CD3 mA were together stained with a cocktail antibody. The expression of tested proteins in CD4 and CD8 T-cells was measured by BD FACSCelesta™ flow cytometer BD Bioscience (San Jose, CA, USA) and analyzed with FlowJo software.

### 2.6. Statistical Analysis

Results for numerical data were expressed as mean ± standard deviation (SD) or median, interquartile range (IQR). Results with proportion were expressed as frequencies and percentages. Independent sample t-tests and Mann–Whitney U Test were used to compare differences between the groups for parametric and non-parametric data, respectively. Fisher’s exact test was used to compare the categorical data. The Wilcoxon signed-rank test was used for comparing the nonparametric data before the first vaccination, 4-weeks and 12-weeks after vaccination in each group. Statistical significance was accepted at the *p*-value < 0.05. All statistical analyses were performed using SPSS version 16 and GraphPad Prism software version 9.4.1 (GraphPad Software, San Diego, CA, USA).

## 3. Results

### 3.1. Study Cohort

There were, in total, 86 healthy elderly participants with controlled underlying diseases, including 21 COPD patients, in our cohort. Nine patients with COPD received a homologous ChAdOx-1 vaccination regimen. One of the COPD patients was lost to follow-up. Therefore, eight COPD patients matched with eight control participants were recruited. The mean ages of COPD and healthy elderly groups were 77.1 ± 6.5 years and 73.5 ± 4.8 years, respectively. There was no significant difference in baseline characteristics between the two groups, including sex, height, body weight, body mass index, and underlying diseases, except for more smoking history in the COPD group. The mean % predicted FEV_1_ in COPD subjects was 69.7 ± 27.7. The detailed data are shown in Table 1. 

### 3.2. Neutralizing Antibody Responses

**Figure 1:**
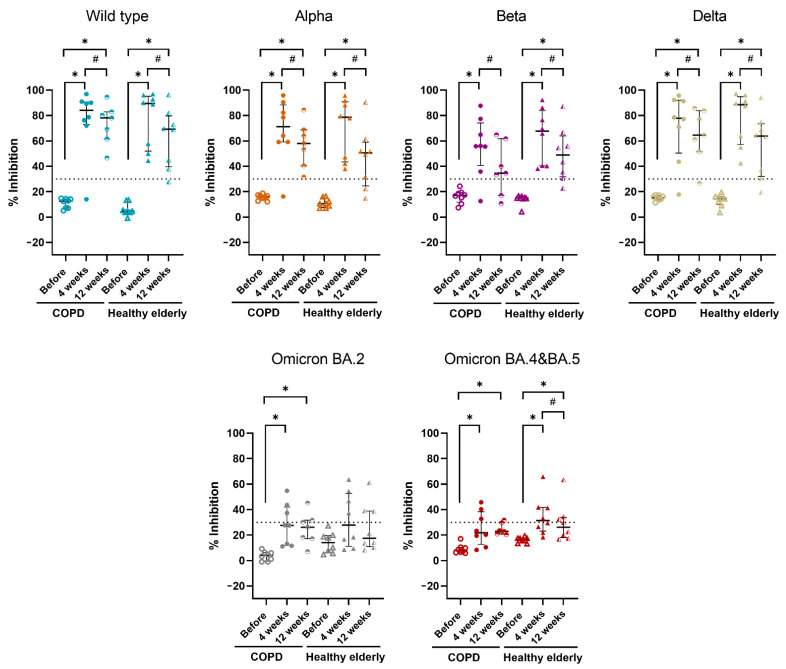
Neutralizing antibodies against SARS-CoV-2 wild-type, Alpha, Beta, Delta, Omicron BA.2, and Omicron BA.4 and BA.5 variants after receiving ChAdOx-1/ChAdOx-1 homologous vaccination. Note: Plasma was obtained from COPD (*n* = 8 or 7) and healthy elderly (*n* = 8) before and at 4 weeks or 12 weeks after the 2nd dose of ChAdOx-1/ChAdOx-1 homologous vaccine. The % inhibition of neutralizing antibodies against wild-type, Alpha, Beta, Delta and Omicron variants are shown. Dot plots exhibit as median with interquartile range which a dot represents each individual. The horizontal lines indicate 30% inhibition threshold level of neutralizing antibody. *, *p*-value < 0.05 compared to before receiving vaccines; #, *p*-value < 0.05 compared to 4 weeks after receiving vaccines using Wilcoxon signed-rank test.

Eight COPD patients with matched healthy controls were tested for NAb as protocol, except for one COPD subject who was lost to follow-up at 12 weeks. The median levels of % inhibition of NAb against the WT, Alpha, Beta, Delta, Omicron BA.2, and Omicron BA.4 and BA.5 before and at 4, 12 weeks after receiving ChAdOx-1/ChAdOx-1 homologous vaccine in COPD and healthy elderly groups are shown in Figure 1 and Table 2. In both groups, after receiving a homologous vaccine for 4 and 12 weeks, the median levels of % inhibition of NAb were significantly higher compared to baseline, except for Beta variants at 12 weeks in the COPD group and Omicron BA.2 variants at 4 and 12 weeks in the healthy elderly group. For 12 weeks after vaccination, the median levels of NAb against all tested VOCs were significantly lower compared to 4 weeks, except for Omicron BA.2 variants in both groups and BA.4 and BA.5 in the COPD group (Figure 1 and Table 2). There was no difference in median levels of % inhibition of NAb against all tested SARS-CoV-2 at each timeline post-vaccination between COPD group and control group (Figure 1 and Table 2). It is worth noting that the NAb against the Omicron variant, both BA.2 and BA.4 and BA.5, were much lower than those of other tested variants. The median levels of % inhibition of NAb against the Omicron variant were lower than 30% inhibition threshold level of neutralizing antibody, except for Omicron BA.4 and BA.5 at 4 weeks post-vaccination in healthy subjects which was slightly higher than the 30% inhibition cutoff (31.3%). 

### 3.3. T-Cell Responses

**Figure 2:**
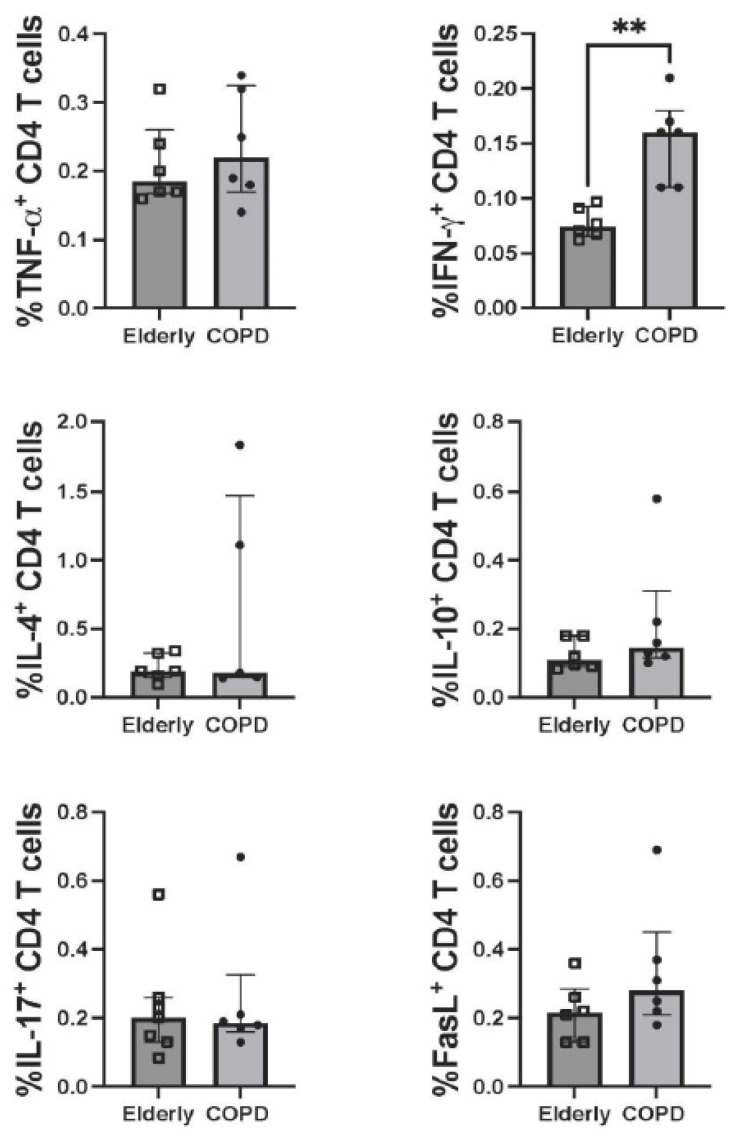
CD4 T-cell responses to pooled peptides of SARS-CoV-2 wild-type spike proteins. Note: PBMCs obtained from COPD (N = 6) and healthy elderly (*n* = 6) at 4 weeks after ChAdOx-1/ChAdOx-1 homologous vaccination were stimulated with pooled peptides of the wild-type spike protein (all functional domains) and analyzed for the expression of molecules of interest using immunofluorescence staining and flow cytometry. The graphs indicate frequency of CD4 T-cells expressing TNF-α, IFN- γ, IL-4, IL-10, IL-17A and FasL. Individual data of each study group are shown. Lines represent the median with the interquartile range. The Mann–Whitney U test was used for comparisons. **, *p* ≤ 0.01.

**Figure 3:**
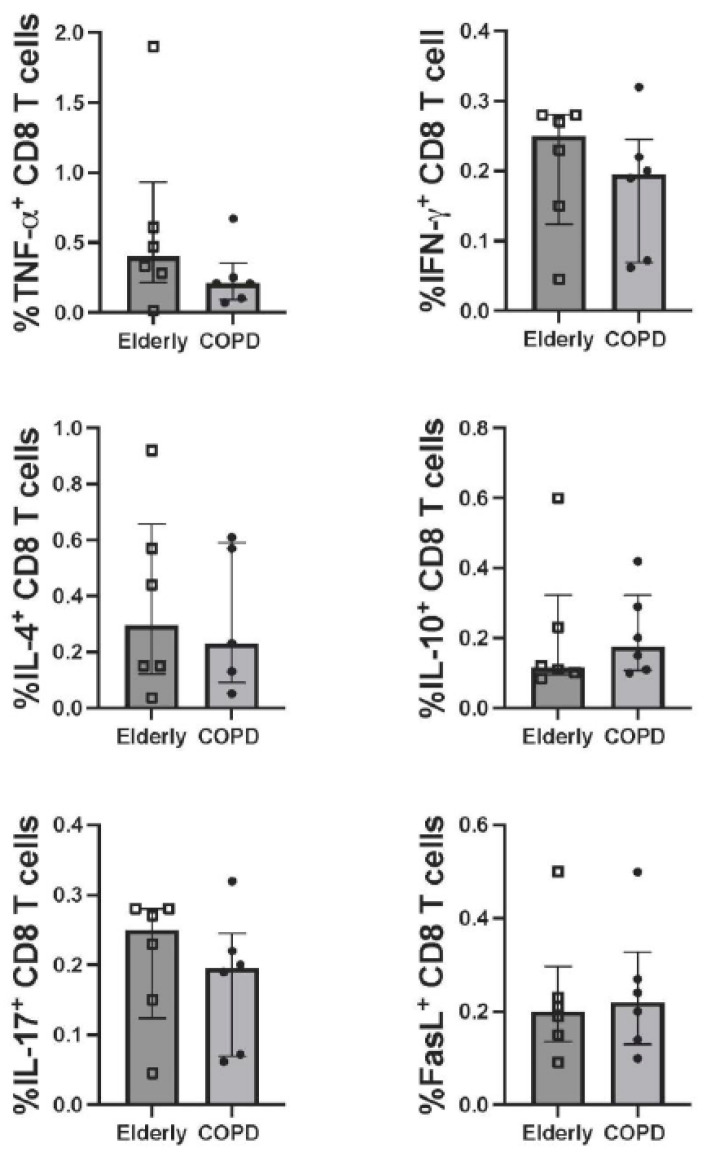
CD8 T-cell responses to pooled peptides of SARS-CoV-2 wild-type spike proteins. Note: PBMCs obtained from COPD (N = 6) and healthy elderly (*n* = 6) at 4 weeks after ChAdOx-1/ChAdOx-1 homologous vaccination were stimulated with pooled peptides of the wild-type spike protein (all functional domains) and analyzed for the expression of molecules of interest using immunofluorescence staining and flow cytometry. The graphs indicate frequency of CD4 T-cells expressing TNF-α, IFN- γ, IL-4, IL-10, IL-17A and FasL. Individual data of each study group are shown. Lines represent the median with the interquartile range. The Mann–Whitney U test was used for comparisons. All data are no statistically significant difference (*p* > 0.05).

Six COPD patients with matched healthy controls were tested for T-cell responses. The SARS-CoV-2 specific CD4 and CD8 T-cell responses at four weeks after receiving the ChAdOx-1/ChAdOx-1 homologous vaccine were compared between COPD and healthy elderly groups. Upon stimulation of PBMCs with peptide pooled of WT S protein, the CD4 and CD8 T-cells producing TNF-α, IFN-γ, IL-4, IL-10, IL-17A, and FasL were determined. The gating strategy of flow cytometric analysis is shown in Supplementary Figure S1. Even though there was no significant difference in antibody production, the number of CD4 T-cells producing IFN-γ in the COPD group was higher than in the healthy elderly group (Figure 2). It was worth noting that the increasing trends, but no statistical significance, of CD4 T-cells producing TNF-α, IL-10, and FasL in the COPD group were also observed. These observations, however, were not detected in CD8 T-cells (Figure 3). 

**Figure 4:**
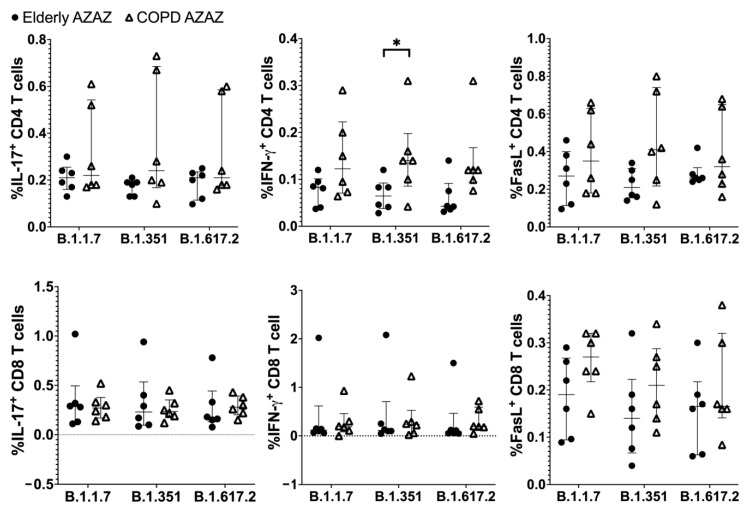
CD4 and CD8 T-cell responses to pooled peptides of spike proteins of SARS-CoV-2 variants. Note: PBMCs obtained from COPD (N = 6) and healthy elderly (*n* = 6) at 4 weeks after ChAdOx-1/ChAdOx-1 homologous vaccination were stimulated with spike peptide pools of B.1.1.7 (Alpha), B.1.351 (Beta), or B.1.617.2 (Delta) mutants and analyzed for the expression of molecules of interest using immunofluorescence staining and flow cytometry. The graphs indicate frequency of CD4 T-cells and CD8 T-cells expressing IL-17A, IFN- γ, and FasL. Individual data of each study group are shown. Lines represent the median with the interquartile range. The Mann–Whitney U test was used for comparisons. *, *p* ≤ 0.05.

T-cell responses to S peptides of SARS-CoV-2 variants were also investigated. PBMCs were stimulated with Alpha, Beta, or Delta variant-specific S peptide pools, and T-cells producing IFN-γ, IL-17 and FasL were determined. As shown in Figure 4, the CD4 T-cell producing IFN-γ in response to S peptides of Beta variant was statistically significantly higher in COPD patients than in the healthy group. The CD4 T-cells producing other tested molecules in response to VOCs were also increased, but without statistical significance, in the COPD group. Again, there was no difference in CD8 T-cell producing the tested markers between COPD patients and healthy elderly (Figure 4). 

## 4. Discussion

COPD patients are associated with an increased risk of severe COVID-19 infection and mortality [1,3]. A systematic review suggested that individuals with COPD should be prioritized for COVID-19 vaccination [12]. However, there are limited data of COVID-19 vaccination on immunogenicity in patients with COPD. Our study focused on the comparison of the induction of NAb against SARS-CoV-2 WT and VOCs (Alpha, Beta, Delta, and Omicron) between vaccinated COPD patients and vaccinated healthy elderly before, and 4 and 12 weeks after, receiving the second dose of ChAdOx-1/ChAdOx-1 homologous vaccine. Moreover, the CD4 and CD8 T-cell responses against S peptides of SARS-CoV-2 four weeks after vaccination were also compared. 

We demonstrated that induction of NAb against the WT, Alpha, Beta, and Delta variants of SARS-CoV-2 was comparable between COPD and healthy elderly groups at 4 and 12 weeks after receiving this vaccine regimen. Our results were supported by the previous findings indicating that immune responses after completing two doses of the COVID-19 vaccine, either with ChAdOx-1 or BNT162b2, were comparable between subjects with COPD and healthy controls [9]. However, our findings were in contrast to the previous studies in the influenza vaccination model which indicated that subjects with COPD had lower antibody response to influenza vaccines than healthy non-COPD controls [4,5,6]. The difference in antibody response observed might come from the different platforms of the vaccine used. The inactivated vaccine was used for the influenza vaccine in the previous study but the adenovirus-vectored COVID-19 vaccine was used in this study. The lower ability to induce immune responses by inactivated vaccines has been suggested and, therefore, affects the antibody responses in influenza-vaccinated COPD subjects. 

In contrast to the Alpha, Beta, and Delta variants, after ChAdOx-1/ChAdOx-1 homologous vaccination, the NAbs against Omicron BA.2 and BA.4 and BA.5 in both COPD and healthy controls were very low. The NAb levels were lower than the 30% inhibition threshold, except those of healthy subjects which were only slightly above the 30% threshold. The inefficient induction of NAb against the Omicron variant was also observed in the healthy elderly after vaccination with COVID-19 vaccines [13]. 

Our study showed that after receiving the ChAdOx-1/ChAdOx-1 homologous vaccine for 4 weeks, the levels of % inhibition of NAb were significantly higher compared to pre-vaccination which were supported by the previous studies [14]. However, our results were different from that study, which recruited younger healthcare workers, on the durability of the antibody. They showed that the NAb levels at one month and three months post-vaccination were indifferent [14]. In contrast, our study showed that at the 12th week, the median levels of NAb were significantly lower compared to the 4th week which indicated a waning of humoral immunity in both COPD and elderly patients. Brockman et al. suggested that the humoral responses to COVID-19 vaccines were significantly weaker in older adults. They demonstrated significant declination of all humoral responses three months after the second dose of mRNA COVID-19 vaccination in older adults [15]. These results were suggestive of faster rate of decline in NAb levels in the elderly population. Therefore, the booster dose of SARS-CoV-2 vaccines may be required in the elderly, both healthy individuals and persons with COPD. 

In addition to NAb, SARS-CoV-2 specific T cells are critical in the immunity to SARS-CoV-2 infection. Both CD4 and CD8 T-cell activation are necessary for the control of infection and are associated with milder diseases [16,17,18]. In this study, the SARS-CoV-2 S peptide-specific CD4 and CD8 T-cell responses at 4 weeks after vaccination of the study groups were compared. We stimulated PBMCs with S peptides derived from WT, Alpha, Beta, and Delta variants and determined intracellular cytokines (i.e., TNF-α, IFN-γ, IL-4, IL-10, and IL-17) and FasL expression. CD4 T-cells are the cells that play a central role in orchestrating adaptive immune responses. Currently, five major CD4 T helper cell (Th) subsets have been described: Th1, Th2, Th17, Treg (T regulatory), and Tfh (follicular T helper) cells. Th1, Th2, Th17, and Treg cells are defined by the production of IFN-γ, IL-4, IL-17, and IL-10, respectively [19]. Our experiments, therefore, monitored the Th1, Th2, Th17, and Treg cell responses after vaccination. FasL is a type-II transmembrane protein expressed on T cells. The FasL-Fas interaction of CD4 or CD8 T cells and target cells has been demonstrated to be involved in T-cell-mediated cytotoxicity [20,21,22]. The investigated cytokines and FasL molecule might indicate the effectiveness of the vaccine in the activation of T-cell responses.

In our study, upon stimulation with WT S peptides (which include all functional domains), CD4 T-cells expressing IFN-γ were higher in the COPD group than in the healthy elderly group. It was worth noting that, although there was no statistically significant difference, the trends in the increase of other tested cytokines and FasL were also demonstrated in the COPD group. However, we did not observe these phenomena in CD8 T-cells in response to the WT S peptides. Furthermore, we found that specific CD4 and CD8 T-cell responses to the S peptides derived from Alpha, Beta, and Delta variants showed the same patterns as those observed with WT S peptide stimulation. Our results suggested that CD4 T-cells primed against the WT S peptides from the immunized vaccines could react to S peptides of SARS-CoV-2 variants. We, therefore, confirmed the cross-reactivity of S peptides derived from WT and the tested VOCs. The T-cell responses against S peptides of the tested VOCs and their WT homologs were not different (Supplementary data Figure S2). The results suggested that, even with an unknown molecular mechanism, the ChAdOx-1/ChAdOx-1 homologous vaccination-induced T helper cells rather than cytotoxic T cells in subjects with COPD. These results are in agreement with the previous report that demonstrated that, after non-typeable *Haemophilus influenzae* protein (NTHi) vaccination, the antigen-specific CD4 T-cell response was induced, but no detectable specific CD8 T-cell responses [23]. In addition, our results were in the line with previous studies showing that, during SARS-CoV-2 infection, CD4 T-cell responses were somewhat larger than those of the CD8 T-cells [1,24]. It had been reported that SARS-CoV-2 infection and disease were controlled by T-cell activation. The strong and early T-cell responses were associated with mild disease [25,26]. However, there was minimal induction of T cells in moderate to severe disease [27] and impairment in T-cell responses in the dead [28]. Nevertheless, the opposite results had been reported that higher activation of T cells was observed in severe disease [29]. SARS-CoV-2 infection and disease were controlled by a fine balance between CD4 and CD8 T-cells. Activation of CD8 T cell responses is associated with effective virus clearance and improved COVID-19 clinical outcomes. However, higher expression of memory CD4 T cells compared to CD8 T cells might contribute to poor clinical outcomes observed in COVID-19 COPD patients [24]. Whether the high induction of CD4 T-cell responses by the COVID-19 vaccine observed in people with COPD contributes to the protection or poor clinical outcomes upon SARS-CoV-2 infection requires elucidation. 

Taken together, we demonstrated that ChAdOx-1/ChAdOx-1 homologous vaccination induced robust NAb production in both COPD patients and healthy elderly, indifferently. However, the induction of SARS-CoV-2 specific CD4 T-cell responses was higher in the COPD group. Our study might support current studies which showed contradictory results to the previous studies and guidelines that COPD subjects were not associated with increased risk of SARS-CoV-2 infection [30], poor prognosis of COVID-19 [31,32], and COVID-19-related deaths [33]. Further immunologic studies are required to clarify whether COPD is really a poor prognostic factor for COVID-19 or not.

There were some strengths of our study. The study was performed in an intermediate-term period for measuring the rate of decline in NAb level. Moreover, the difference from the previous studies which enrolled younger subjects as controls, the age- and sex-matched healthy elderly subjects was included as a control group in this study. The limitation of our study was a small sample size because it was a preliminary study. However, this study was valuable in demonstrating the immunogenicity of the SARS-CoV-2 vaccine, particularly the T-cell responses, in this vulnerable group of patients. Our small study will inspire the other researchers to conduct the future studies with a larger sample size to confirm these results and having stronger evidences for prevention of the next possible emerging infection. In the study of T-cell responses, upon vaccination, the changes in T-cell activation markers and multifunctional T-cell responses were also not monitored. 

## 5. Conclusions

The improvement of NAb levels at 4 and 12 weeks after receiving ChAdOx-1/ChAdOx-1 homologous vaccination against the WT, Alpha, Beta, and Delta variants of SARS-CoV-2 were comparable between COPD and healthy elderly. After receiving this homologous vaccination for 4 and 12 weeks, the levels of NAb were significantly higher compared to baseline. However, at 12 weeks the levels of % inhibition of NAb were significantly declining from 4 weeks after receiving vaccines. The levels of NAb against the Omicron variant, however, were lower than those of the WT, Alpha, Beta, and Delta variants. This homologous vaccination regimen, interestingly, induced specific CD4 T-cell responses higher in COPD than those of the healthy elderly group.

## Figures and Tables

**Table 1:** Demographic and clinical characteristics of participants.

Characteristics	COPD (N = 8)	Healthy Elderly (N = 8)	*p*-Value
Age (years)	77.1 ± 6.5	73.5 ± 4.8	0.224
Male (sex)	7 (87.5)	7 (87.5)	1.000
Height (cm)	159.9 ± 7.2	165.1 ± 9.8	0.240
Body weight (kg)	54.9 ± 10.1	62.0 ± 8.9	0.157
Body mass index (kg/m^2^)	21.6 ± 5.0	22.8 ± 2.9	0.593
Underlying diseases			0.721
Cardiovascular	2 (25.0)	2 (25.0)	
Metabolic	0 (0.0)	1 (12.5)	
Cardiovascular + Metabolic	2 (25.0)	1 (12.5)	
None	4 (50.0)	4 (50.0)	
Smoking status			0.026
Non-smoker	0 (0.0)	5 (62.5)	
Ex-smoker	8 (100.0)	3 (37.5)	
Smoking pack-year (Median, IQR)	45.0 (25.3, 50.0)	0.0 (0.0, 30.0)	0.012
Spirometry results		N.A.	
FVC	2.49 ± 0.58		
% predicted of FVC	96.9 ± 23.7		
FEV_1_	1.38 ± 0.45		
% predicted of FEV_1_	69.7 ± 27.7		
Ratio of FEV_1_/FVC	54.4 ± 9.9		
Inhaled medication for COPD		N.A.	
LAMA	1 (12.5)		
LABA + LAMA	1 (12.5)		
ICS + LABA	1 (12.5)		
ICS + LAMA	1 (12.5)		
ICS + LABA + LAMA	4 (50.0)		

Note: Data are presented as mean ± SD or *n* (%) or otherwise stated. Abbreviations: FEV_1_, forced expiratory volume in the first second; FVC, forced vital capacity; ICS, inhaled corticosteroids; LAMA, Long-acting muscarinic antagonists; LABA, long-acting beta2-agonists.

**Table 2:** Levels of % inhibition of neutralizing antibodies against the wild-type, Alpha, Beta, Delta, and Omicron variants of SARS-CoV-2 before and at 4 and 12 weeks after receiving ChAdOx-1/ChAdOx-1 homologous vaccination.

	COPD			Healthy Elderly		
	Before (*n* = 8)	4 Weeks (*n* = 8)	12 Weeks (*n* = 7)	Before (*n* = 8)	4 Weeks (*n* = 8)	12 Weeks (*n* = 8)
**Wild-type**	12.6 (7.1, 14.3)	84.0 * (73.2, 90.7)	78.1 *^,#^ (61.5, 82.9)	4.7 (3.7, 11.2)	89.4 * (51.9, 95.2)	69.2 *^,#^ (39.5, 79.7)
**Alpha** **(B.1.1.7)**	15.9 (13.2, 17.1)	71.2 * (59.1, 88.4)	57.9 *^,#^ (40.3, 68.7)	10.5 (7.4, 15.1)	78.6 * (43.4, 90.8)	50.5 *^,#^ (24.5, 59.0)
**Beta** **(B.1.351)**	17.0 (11.5, 19.1)	55.9 * (40.7, 74.2)	34.6 ^#^ (16.9, 61.7)	14.9 (14.6, 16.1)	67.7 * (40.3, 83.7)	48.9 *^,#^ (31.7, 63.9)
**Delta (B.1.617.2)**	15.2 (13.7, 16.9)	77.8 * (50.4, 91.9)	64.5 *^,#^ (51.3, 83.8)	14.5 (9.7, 15.9)	88.6 * (57.2, 94.8)	63.8 *^,#^ (31.9, 73.4)
**Omicron BA.2**	3.9 (−0.4, 6.2)	27.6 * (11.9, 41.9)	26.2 * (17.1, 31.7)	14.0 (6.2, 19.6)	27.8 (11.1, 52.6)	17.5 (10.9, 38.8)
**Omicron BA.4&BA.5**	7.9 (6.3, 10.3)	21.8 * (12.7, 38.3)	22.8 * (20.7, 29.7)	16.5 (13.9, 17.5)	31.3 * (23.0, 41.7)	26.2 *^,#^ (17.9, 33.9)

Note: Data are presented as median (interquartile range, IQR); *, *p*-value < 0.05 compared to before receiving vaccines; ^#^, *p*-value < 0.05 compared to 4 weeks after receiving vaccines. Abbreviations: COPD, chronic obstructive pulmonary disease.

## Data Availability

The data that support the findings of this study are available on request from the corresponding author.

## Supplementary Materials

The following are available online at https://www.mdpi.com/article/10.3390/vaccines10122176/s1, Figure S1: Flow cytometry and gating strategy. Figure S2: CD4 and CD8 T-cell responses to pooled spike peptides of SARS-CoV-2 variants and their wild-type peptide homologues.
