# Supplementary material for: Neutralizing Antibody and T-Cell Responses against SARS-CoV-2 Wild-Type and Variants of Concern in Chronic Obstructive Pulmonary Disease Subjects after ChAdOx-1/ChAdOx-1 Homologous Vaccination: A Preliminary Study
Source: Vaccines (Basel). 2022 Dec 18;10(12):2176. doi: 10.3390/vaccines10122176 (PMC9781239; doi:10.3390/vaccines10122176)
Supplement: Supplementary file 1 [file vaccines-10-02176-s001.zip › vaccines-2056797-supplementary.pdf]

Supplement Figure S1

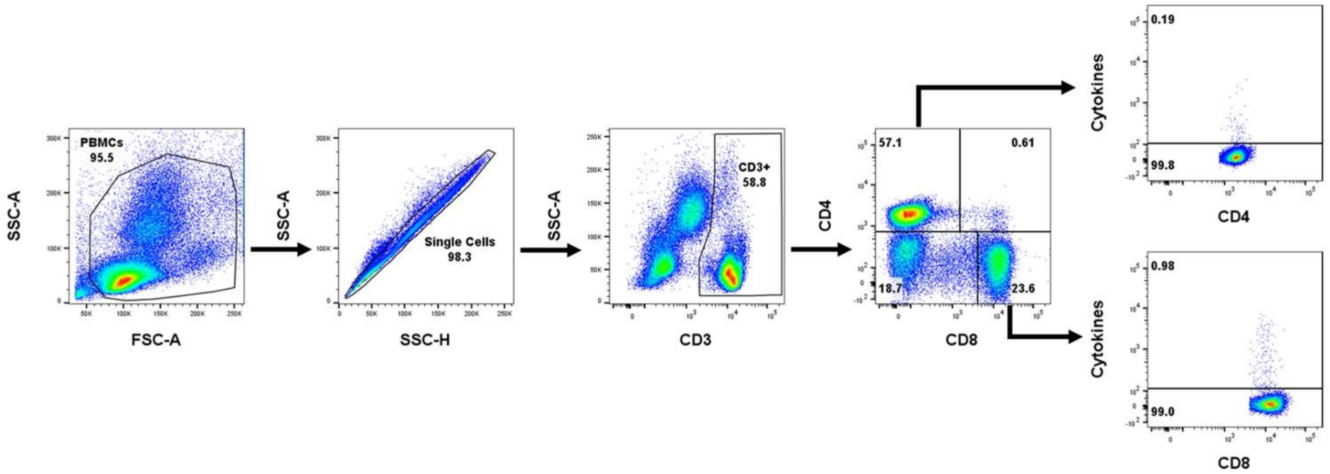

Figure S1. Flow cytometry and gating strategy. PBMCs were stimulated with the SARS-CoV-2 peptide pool of spike proteins, and indicated cytokines and FASL expression levels were determined by flow cytometry. The gating strategies are shown. The size (FSC-A) and granularity (SSC-A) of PBMCs were plotted and gated as indicated. The gated cells were represented in an SSC-H vs. SSC-A dot plot to eliminate doublets. CD3 T cells were gated by plotting CD3 staining vs. SSC-A. CD4 T cells and CD8 T cells were gated from CD3 T cells by plotting CD8 vs. CD4 staining. The CD4 T cells and CD8 T cells were plotted against CD4 or CD8 staining and molecule of interest. The frequency in each population was determined by FlowJo software.

Supplement Figure S2

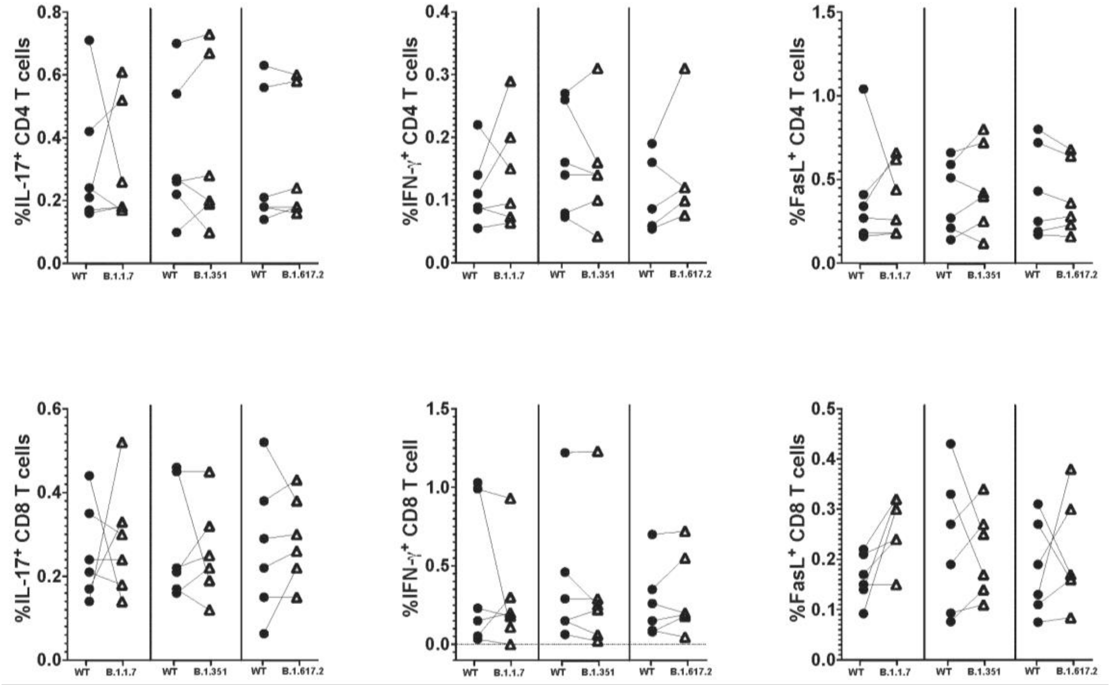

Figure S2. CD4 and CD8 T cell responses to pooled spike peptides of SARS-CoV-2 variants and their wild type peptide homologues. PBMCs of COPD patients (n=6) at 4 weeks after ChAdOx-1/ChAdOx-1 homologous vaccination were stimulated with spike peptide pools of B.1.1.7 (Alpha), B.1.351 (Beta), or B.1.617.2 (Delta) mutants or their WT peptide homologue. By immunofluorescence staining and flow cytometry, CD4 T cells or CD8 T cells were gated to determine the frequency of cells expressing IL-17, IFN-γ and FasL. Individual data in each tested condition are presented. The Wilcoxon matched pairs signed-ranks test was used for comparison. No significant difference ($p > 0.05$) between mutants and their homologous WT peptides was observed.
